# 1,1′,4,5-Tetra­hydro­tri­spiro­[1,3,2-di­aza­phosphole-2,2′-[1,3,5,2,4,6]tri­aza­triphosphinine-4′,6′′-dibenzo[*d*,*f*][1,3,2]dioxaphosphepine-6′,6′′′-dibenzo[*d*,*f*][1,3,2]dioxaphosphepine] acetone monosolvate

**DOI:** 10.1107/S1600536813023830

**Published:** 2013-08-31

**Authors:** Krystal R. Fontenot, Michael W. Easson, Frank R. Fronczek, Brian D. Condon

**Affiliations:** a United States Department of Agriculture Cotton Chemistry and Utilization, 1100 Robert E. Lee Blvd, New Orleans, LA 70124, USA; b Department of Chemistry, Louisiana State University, Baton Rouge, LA 70803, USA

## Abstract

The title compound, C_26_H_22_N_5_O_4_P_3_·C_3_H_6_O, has been achieved in a two-step synthesis that does not require chromatography. This mol­ecule contains a seven-membered spiro­cyclic ring at two P-atom positions and a five-membered ring containing new P—N bonds at the other P-atom position. Endocyclic torsion angles about the central biphenyl C—C bonds are −41.5 (3) and −44.4 (3)°, and P—N bonds of the central P_3_N_3_ ring are within the range 1.5665 (17)–1.6171 (17) Å, while the P—O distances are in the range 1.5940 (14)–1.6041 (14) Å. One N—H group makes an inter­molecular N—H⋯N hydrogen bond, forming centrosymmetric dimers, while the other N—H group makes an N—H⋯O hydrogen bond to the acetone solvent mol­ecule. The crystal was a two-component non-merohedral twin with ratio 0.811/0.189.

## Related literature

For phosphazene-based flame retardants, see: Bakos *et al.* (1982 ▶); Drews & Barker (1985 ▶). For related structures, bond angles and lengths, see: Allcock (1972 ▶); Ciftci *et al.* (2013 ▶). For the geometry of phosphazene rings, see: Olthof (1969 ▶); Barclay *et al.* (2002 ▶). For the synthesis, see: Allen (1991 ▶); Carriedo *et al.* (1996 ▶). For related structures, see: Chandrasekhar *et al.* (2007 ▶; 2011 ▶; 2012 ▶); Harmjanz *et al.* (2004 ▶). For graph-set analysis, see: Etter (1990 ▶). For ring asymmetry parameters, see: Duax *et al.* (1976 ▶).
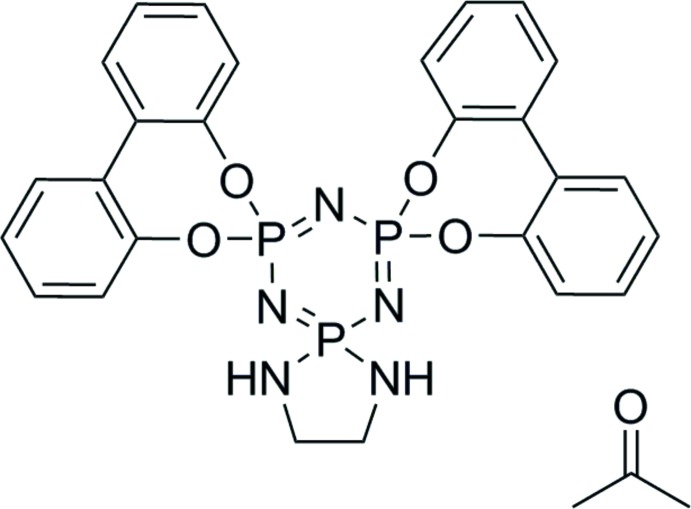


## Experimental

### 

#### Crystal data

C_26_H_22_N_5_O_4_P_3_·C_3_H_6_O
*M* *_r_* = 619.47
Monoclinic, 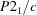

*a* = 9.4901 (9) Å
*b* = 22.9466 (19) Å
*c* = 13.1776 (13) Å
β = 97.978 (6)°
*V* = 2841.9 (5) Å^3^
*Z* = 4
Cu *K*α radiation
μ = 2.34 mm^−1^
*T* = 100 K
0.19 × 0.12 × 0.03 mm

#### Data collection

Bruker Kappa APEXII DUO CCD diffractometer
Absorption correction: multi-scan (*TWINABS*; Sheldrick, 2004 ▶) *T*_min_ = 0.664, *T*_max_ = 0.933
33208 measured reflections
5065 independent reflections
4276 reflections with *I* > 2σ(*I*)
*R*_int_ = 0.051

#### Refinement

*R*[*F* ^2^ > 2σ(*F* ^2^)] = 0.037
*wR*(*F* ^2^) = 0.102
*S* = 1.08
5065 reflections
387 parameters
H atoms treated by a mixture of independent and constrained refinement
Δρ_max_ = 0.40 e Å^−3^
Δρ_min_ = −0.39 e Å^−3^

### 

Data collection: *APEX2* (Bruker, 2011 ▶); cell refinement: *SAINT* (Bruker, 2011 ▶); data reduction: *SAINT*; program(s) used to solve structure: *SHELXS97* (Sheldrick, 2008 ▶); program(s) used to refine structure: *SHELXL97* (Sheldrick, 2008 ▶); molecular graphics: *ORTEP-3 for Windows* (Farrugia, 2012 ▶); software used to prepare material for publication: *SHELXL97*.

## Supplementary Material

Crystal structure: contains datablock(s) I, New_Global_Publ_Block. DOI: 10.1107/S1600536813023830/jj2173sup1.cif

Structure factors: contains datablock(s) I. DOI: 10.1107/S1600536813023830/jj2173Isup2.hkl

Additional supplementary materials:  crystallographic information; 3D view; checkCIF report

## Figures and Tables

**Table 1:** Hydrogen-bond geometry (Å, °)

*D*—H⋯*A*	*D*—H	H⋯*A*	*D*⋯*A*	*D*—H⋯*A*
N4—H4*N*⋯O5	0.82 (3)	2.22 (3)	2.991 (2)	158 (2)
N5—H5*N*⋯N3^i^	0.83 (3)	2.53 (3)	3.285 (2)	151 (2)

Symmetry code: (i) 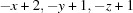.

**Fig. 1.**
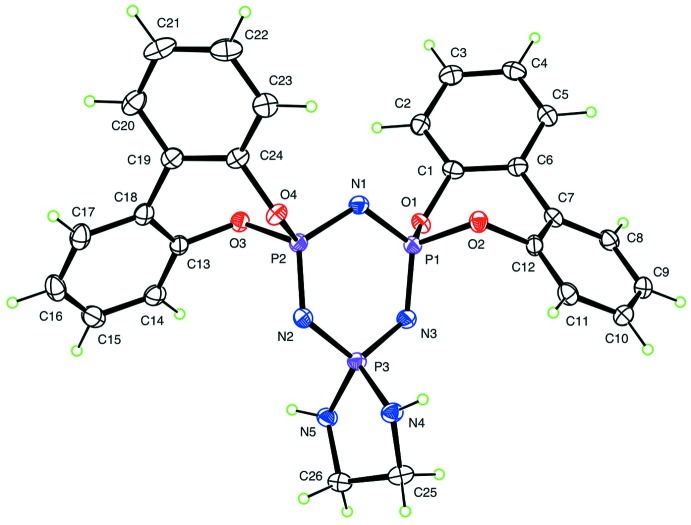
The molecular structure of the title compound with atomic numbering scheme and 50% ellipsoids. The solvent molecule is not shown.

**Fig. 2.**
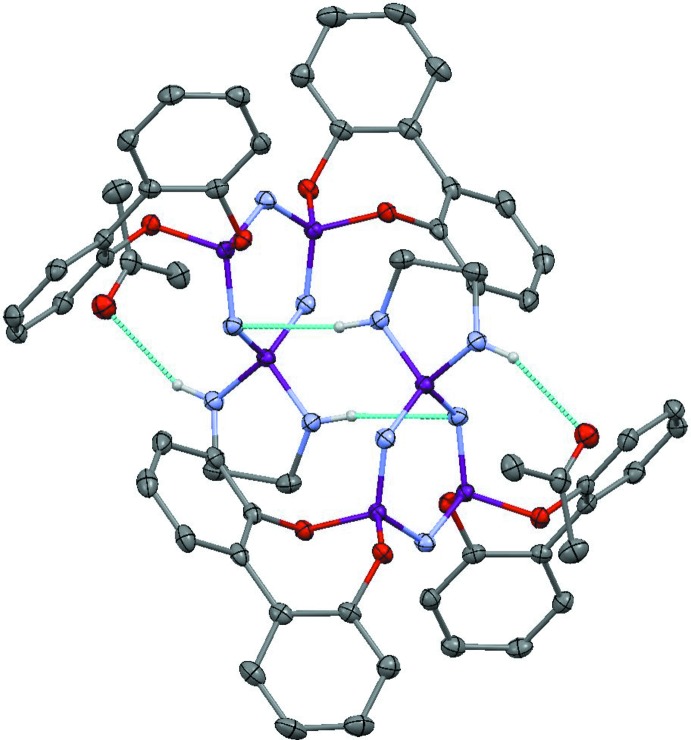
Hydrogen bonding, illustrating the centrosymmetic dimer about 1, 1/2, 1/2 and the two acetone acceptors. H atoms on C are not shown.

## supplementary crystallographic information

### 1. Comment

The non-halogenated title compound contains two key elements phosphorus and nitrogen, which are incorporated into the backbone moiety of the molecule to achieve flame retardant properties (Bakos *et al.*, 1982). Studies have shown that an increase in the number of phosphorus (P), nitrogen (N), or P—N bonds leads to improved flame retardancy (Drews & Barker, 1985). The substitution of two biphenol groups and ethylenediamine onto phosphazene was achieved through nucleophilic substitution in high yield. This compound was applied to cotton fabric and has shown promising preliminary results as a potential flame retardant.

The proposed efficacy and novelty of this flame retardant is based on the design of phosphorus (P3) surrounded by four nitrogen (N2, N3, N4, N5) atoms, thereby increasing the number of P—N bonds in the molecule. Incorporation of the 2,2'-biphenol moiety at P1 and P2 promotes a seven membered spirocyclic structure with endocyclic torsion angles about the central biphenol C—C bonds being nearly equal, -44.4 (3)° for C1/C6/C7/C12 and -41.5 (3)° for C13/C18/C19/C24. Both seven-membered rings have twist conformations with the P atom on the local twofold axis; however the deviation from C_2_ local symmetry is substantial for both. The asymmetry parameter (Duax *et al.*, 1976) is 14.83 (13)° for the ring containing P1 and 18.44 (14)° for that containing P2.

The utilization of ethylenediamine at the P3 position forms a five-membered ring with new P—N bonds, which lies roughly perpendicular (dihedral angle 81.42 (4)°) to the phosphazene ring. The conformation of the 5-membered ring is closest to an envelope with C25 at the flap position. C25 lies 0.554 (2) Å out of the plane of the other four atoms, and the C_s_ asymmetry parameter is 3.44 (17)°. The N—P—N bonds have angles of 94.47 (9)° to 113.64 (9)°, which is lower than typical angles of ~118° (Allcock, 1972). The narrowing of the angle may be due to van der Waals repulsions from the four N atoms surrounding P3 (Allcock, 1972). It has been reported that N—P—N bonds with two bulky phenyl groups on P have a narrowed angle of 115° due to mutual repulsions form the bulky phenyl group (Allcock, 1972). The phosphazene ring itself is slightly nonplanar, having a boat distortion, with P2 lying 0.1982 (4) Å and N3 lying 0.161 (2) Å on the same side of the plane defined by P1, P3, N1, and N2. The phosphazene ring has P—N bond lengths ranging from 1.5665 (17) to 1.6171 (17) Å. These values are typical for phosphazene rings (Barclay *et al.* 2002; Olthof, 1969). This molecule has similar bond angles and bond lengths, when substituted with the same or comparable spiro molecules, as found in the literature (Allcock, 1972, Ciftci *et al.*, 2013). Overall, the molecule has approximate C_2_ symmetry, as seen in Fig. 1.

Both NH groups donate intermolecular hydrogen bonds, as shown in Fig. 2. The N5—H···N3 (at 2 - *x*, 1 - *y*, 1 - *z*) bond leads to centrosymmetric hydrogen-bonded dimers, with graph set *R*^2^_2_(8) (Etter, 1990), while N4 donates a hydrogen bond to acetone O. The acetone molecule forms two C—H···O hydrogen bonds with the phosphazene molecule, one as a donor and the other as an acceptor (Table 1).

### 2. Experimental

All reagent grade chemicals were purchased from Sigma Aldrich and were used without further purification. ESI Mass Spectra were recorded on an Agilent Technologies 6520 Accurate Mass Q-TOF LC/MS. NMR spectra were recorded using a Varian 400MHZ spectrometer. ^1^H and ^13^C are given in δ relative to TMS and ^31^P is given δ relative to external 85% aqueous H_3_PO_4_.

2,2-Dichloro-4,4,6,6-bis[spiro(2,2'-dioxy-1'1"-biphenyl)] Cyclotriphosphazene (1) was synthesized as previously reported in the literature (Allen, 1991; Carriedo *et al.* 1996).

A 3-neck round bottom flask was equipped with an argon inlet, an addition funnel, and a stopper. (1) was allowed to stir in CH_2_C1_2_ until completely dissolved. A solution of ethylenediamine in CH_2_C1_2_ was added drop-wise to the round bottom flask at 0°C and allowed to warm to room temperature. The reaction was allowed to stir for 24 h. Thin Layer Chromatography (TLC) was used to follow the reaction using Hexanes/ CH_2_C1_2_ (50:50) and DCM/MeOH (90:10). Purification was achieved by gently extracting the organic layer with warm distilled water. The organic layer was dried by anhydrous sodium sulfate and concentrated. The product (2) obtained was a white solid with a yield of 84%. The compound was tested by ESI-MS and ^1^H, ^13^C, ^31^P –NMR using Acetone-d_6_. Crystals of (2) were grown in an NMR tube from acetone.

### 3. Refinement

H atoms on C were placed in idealized positions with C—H distances 0.95 - 0.99 Å and thereafter treated as riding, with a torsional parameter refined for each methyl group. Coordinates of the NH hydrogen atoms were refined isotropically. *U*_iso_ for H were assigned as 1.2 times *U*_eq_ of the attached atoms (1.5 for methyl).

### Crystal data

**Table:**

C_26_H_22_N_5_O_4_P_3_·C_3_H_6_O	*F*(000) = 1288
*M**_r_* = 619.47	*D*_x_ = 1.448 Mg m^−^^3^
Monoclinic, *P*2_1_/*c*	Cu *K*α radiation, λ = 1.54184 Å
Hall symbol: -P 2ybc	Cell parameters from 3314 reflections
*a* = 9.4901 (9) Å	θ = 3.9–67.8°
*b* = 22.9466 (19) Å	µ = 2.34 mm^−^^1^
*c* = 13.1776 (13) Å	*T* = 100 K
β = 97.978 (6)°	Plate, colorless
*V* = 2841.9 (5) Å^3^	0.19 × 0.12 × 0.03 mm
*Z* = 4	

### Data collection

**Table:**

Bruker Kappa APEXII DUO CCD diffractometer	5065 independent reflections
Radiation source: IµS microfocus	4276 reflections with *I* > 2σ(*I*)
QUAZAR multilayer optics monochromator	*R*_int_ = 0.051
φ and ω scans	θ_max_ = 68.7°, θ_min_ = 3.9°
Absorption correction: multi-scan (TWINABS; Sheldrick, 2004)	*h* = −11→11
*T*_min_ = 0.664, *T*_max_ = 0.933	*k* = 0→27
33208 measured reflections	*l* = 0→15

### Refinement

**Table:**

Refinement on *F*^2^	Primary atom site location: structure-invariant direct methods
Least-squares matrix: full	Secondary atom site location: difference Fourier map
*R*[*F*^2^ > 2σ(*F*^2^)] = 0.037	Hydrogen site location: inferred from neighbouring sites
*wR*(*F*^2^) = 0.102	H atoms treated by a mixture of independent and constrained refinement
*S* = 1.08	*w* = 1/[σ^2^(*F*_o_^2^) + (0.0595*P*)^2^ + 0.7869*P*] where *P* = (*F*_o_^2^ + 2*F*_c_^2^)/3
5065 reflections	(Δ/σ)_max_ = 0.001
387 parameters	Δρ_max_ = 0.40 e Å^−^^3^
0 restraints	Δρ_min_ = −0.39 e Å^−^^3^

### Special details

**Table:**

Experimental. The sample was a multiple crystal ("twin") with two components. The second domain was rotated from first domain by 2.6 degrees about reciprocal axis -0.141 1.000 0.976 and real axis -0.085 0.341 1.000The twin law to convert *hkl* from first to this domain (*SHELXL* TWIN matrix): 1.002 - 0.016 0.017, 0.096 0.999 0.015, -0.032 - 0.001 0.996
Geometry. All e.s.d.'s (except the e.s.d. in the dihedral angle between two l.s. planes) are estimated using the full covariance matrix. The cell e.s.d.'s are taken into account individually in the estimation of e.s.d.'s in distances, angles and torsion angles; correlations between e.s.d.'s in cell parameters are only used when they are defined by crystal symmetry. An approximate (isotropic) treatment of cell e.s.d.'s is used for estimating e.s.d.'s involving l.s. planes.
Refinement. Refinement of *F*^2^ against ALL reflections, using a TWIN4 *hkl* file prepared by TWINABS (Sheldrick, 2004).

### Fractional atomic coordinates and isotropic or equivalent isotropic displacement parameters (Å^2^)

**Table:**

	*x*	*y*	*z*	*U*_iso_*/*U*_eq_	
P1	0.86867 (5)	0.57153 (2)	0.27605 (4)	0.01501 (13)	
P2	0.70274 (5)	0.47853 (2)	0.30698 (4)	0.01500 (13)	
P3	0.79507 (5)	0.55177 (2)	0.47261 (4)	0.01498 (13)	
O1	1.02561 (15)	0.56625 (6)	0.24629 (11)	0.0169 (3)	
O2	0.80895 (14)	0.62781 (6)	0.21219 (11)	0.0171 (3)	
O3	0.77860 (14)	0.41639 (6)	0.30107 (11)	0.0165 (3)	
O4	0.54174 (14)	0.46339 (6)	0.26346 (11)	0.0174 (3)	
N1	0.77084 (18)	0.51945 (7)	0.22950 (13)	0.0173 (4)	
N2	0.69897 (18)	0.49976 (7)	0.41955 (13)	0.0178 (4)	
N3	0.89141 (18)	0.58162 (7)	0.39485 (13)	0.0172 (4)	
N4	0.70206 (19)	0.59910 (8)	0.52986 (14)	0.0190 (4)	
H4N	0.659 (3)	0.6247 (11)	0.4945 (19)	0.023*	
N5	0.89724 (19)	0.53234 (8)	0.57900 (13)	0.0182 (4)	
H5N	0.921 (3)	0.4976 (11)	0.5831 (19)	0.022*	
C1	1.0620 (2)	0.58150 (9)	0.14985 (16)	0.0175 (4)	
C2	1.1120 (2)	0.53795 (9)	0.09149 (16)	0.0195 (4)	
H2	1.1120	0.4985	0.1134	0.023*	
C3	1.1619 (2)	0.55245 (9)	0.00081 (17)	0.0231 (5)	
H3	1.1962	0.5228	−0.0399	0.028*	
C4	1.1620 (2)	0.61020 (10)	−0.03067 (16)	0.0233 (5)	
H4	1.1973	0.6201	−0.0925	0.028*	
C5	1.1105 (2)	0.65351 (9)	0.02810 (16)	0.0205 (4)	
H5	1.1096	0.6928	0.0054	0.025*	
C6	1.0600 (2)	0.64004 (9)	0.12034 (15)	0.0170 (4)	
C7	1.0101 (2)	0.68643 (8)	0.18533 (15)	0.0174 (4)	
C8	1.0836 (2)	0.73950 (9)	0.20275 (16)	0.0203 (4)	
H8	1.1679	0.7460	0.1730	0.024*	
C9	1.0346 (2)	0.78235 (9)	0.26264 (16)	0.0225 (5)	
H9	1.0854	0.8180	0.2735	0.027*	
C10	0.9119 (2)	0.77390 (9)	0.30718 (16)	0.0218 (4)	
H10	0.8794	0.8035	0.3486	0.026*	
C11	0.8369 (2)	0.72215 (9)	0.29101 (16)	0.0205 (4)	
H11	0.7523	0.7159	0.3206	0.025*	
C12	0.8877 (2)	0.67975 (8)	0.23097 (16)	0.0175 (4)	
C13	0.7274 (2)	0.37086 (8)	0.35739 (16)	0.0170 (4)	
C14	0.8068 (2)	0.35465 (9)	0.44930 (16)	0.0207 (4)	
H14	0.8913	0.3751	0.4752	0.025*	
C15	0.7602 (3)	0.30792 (9)	0.50272 (17)	0.0265 (5)	
H15	0.8124	0.2964	0.5663	0.032*	
C16	0.6377 (2)	0.27796 (9)	0.46347 (18)	0.0265 (5)	
H16	0.6068	0.2458	0.5000	0.032*	
C17	0.5602 (2)	0.29493 (9)	0.37092 (18)	0.0234 (5)	
H17	0.4765	0.2741	0.3446	0.028*	
C18	0.6038 (2)	0.34235 (8)	0.31581 (16)	0.0182 (4)	
C19	0.5226 (2)	0.36114 (9)	0.21647 (16)	0.0182 (4)	
C20	0.4629 (2)	0.32016 (9)	0.14425 (18)	0.0246 (5)	
H20	0.4762	0.2798	0.1586	0.029*	
C21	0.3846 (2)	0.33719 (11)	0.05215 (19)	0.0296 (5)	
H21	0.3440	0.3086	0.0047	0.036*	
C22	0.3657 (2)	0.39597 (11)	0.02949 (18)	0.0291 (5)	
H22	0.3130	0.4077	−0.0338	0.035*	
C23	0.4239 (2)	0.43759 (10)	0.09950 (17)	0.0237 (5)	
H23	0.4115	0.4779	0.0845	0.028*	
C24	0.5001 (2)	0.41980 (9)	0.19118 (16)	0.0178 (4)	
C25	0.7808 (2)	0.61684 (9)	0.62821 (16)	0.0233 (5)	
H25A	0.7156	0.6317	0.6748	0.028*	
H25B	0.8514	0.6475	0.6188	0.028*	
C26	0.8542 (2)	0.56094 (9)	0.67000 (16)	0.0213 (4)	
H26A	0.9382	0.5698	0.7211	0.026*	
H26B	0.7880	0.5360	0.7024	0.026*	
O5	0.53486 (16)	0.66469 (7)	0.35745 (12)	0.0258 (3)	
C27	0.4623 (2)	0.62727 (9)	0.30959 (17)	0.0220 (5)	
C28	0.3829 (2)	0.58239 (9)	0.36180 (18)	0.0255 (5)	
H28A	0.4119	0.5846	0.4360	0.038*	
H28B	0.4045	0.5435	0.3373	0.038*	
H28C	0.2805	0.5897	0.3462	0.038*	
C29	0.4499 (3)	0.62433 (11)	0.19530 (18)	0.0307 (5)	
H29A	0.4922	0.6594	0.1695	0.046*	
H29B	0.3493	0.6219	0.1661	0.046*	
H29C	0.5002	0.5898	0.1754	0.046*	

### Atomic displacement parameters (Å^2^)

**Table:**

	*U*^11^	*U*^22^	*U*^33^	*U*^12^	*U*^13^	*U*^23^
P1	0.0158 (3)	0.0150 (2)	0.0145 (3)	−0.00015 (19)	0.00280 (19)	0.00056 (18)
P2	0.0159 (3)	0.0140 (2)	0.0153 (3)	−0.00058 (19)	0.00309 (19)	−0.00092 (18)
P3	0.0156 (3)	0.0156 (2)	0.0139 (3)	0.00053 (19)	0.00267 (19)	−0.00072 (18)
O1	0.0168 (7)	0.0184 (7)	0.0161 (7)	0.0007 (5)	0.0041 (6)	0.0038 (5)
O2	0.0156 (7)	0.0159 (7)	0.0194 (7)	−0.0013 (5)	0.0007 (6)	0.0012 (5)
O3	0.0165 (7)	0.0152 (6)	0.0183 (7)	−0.0007 (5)	0.0041 (5)	−0.0009 (5)
O4	0.0157 (7)	0.0159 (6)	0.0206 (8)	0.0000 (5)	0.0024 (6)	−0.0040 (6)
N1	0.0196 (9)	0.0177 (8)	0.0151 (9)	−0.0020 (7)	0.0042 (7)	−0.0015 (6)
N2	0.0195 (9)	0.0173 (8)	0.0173 (9)	−0.0020 (7)	0.0051 (7)	0.0005 (7)
N3	0.0181 (9)	0.0174 (8)	0.0158 (9)	−0.0023 (7)	0.0016 (7)	−0.0005 (7)
N4	0.0203 (9)	0.0193 (8)	0.0176 (9)	0.0041 (7)	0.0030 (7)	−0.0017 (7)
N5	0.0204 (9)	0.0187 (8)	0.0154 (9)	0.0028 (7)	0.0019 (7)	0.0007 (7)
C1	0.0141 (10)	0.0234 (10)	0.0151 (10)	−0.0017 (8)	0.0029 (8)	0.0003 (8)
C2	0.0189 (10)	0.0177 (9)	0.0215 (11)	−0.0003 (8)	0.0013 (8)	−0.0007 (8)
C3	0.0216 (11)	0.0263 (11)	0.0212 (11)	0.0020 (9)	0.0025 (9)	−0.0039 (9)
C4	0.0239 (11)	0.0307 (11)	0.0160 (11)	−0.0022 (9)	0.0051 (9)	0.0007 (9)
C5	0.0215 (11)	0.0203 (10)	0.0198 (11)	−0.0004 (8)	0.0031 (8)	0.0031 (8)
C6	0.0151 (10)	0.0193 (10)	0.0167 (10)	−0.0017 (8)	0.0025 (8)	−0.0015 (8)
C7	0.0191 (10)	0.0181 (9)	0.0148 (10)	0.0008 (8)	0.0016 (8)	0.0033 (8)
C8	0.0225 (11)	0.0207 (10)	0.0184 (11)	−0.0024 (8)	0.0054 (8)	0.0034 (8)
C9	0.0307 (12)	0.0159 (9)	0.0209 (11)	−0.0046 (9)	0.0031 (9)	0.0009 (8)
C10	0.0286 (11)	0.0171 (10)	0.0201 (11)	0.0019 (9)	0.0051 (9)	−0.0019 (8)
C11	0.0199 (10)	0.0204 (10)	0.0212 (11)	0.0029 (8)	0.0036 (8)	0.0038 (8)
C12	0.0198 (10)	0.0132 (9)	0.0186 (10)	−0.0013 (8)	−0.0004 (8)	0.0023 (7)
C13	0.0207 (10)	0.0139 (9)	0.0182 (10)	0.0005 (8)	0.0091 (8)	−0.0011 (8)
C14	0.0228 (11)	0.0184 (9)	0.0213 (11)	0.0052 (8)	0.0044 (9)	−0.0030 (8)
C15	0.0379 (13)	0.0220 (10)	0.0208 (11)	0.0116 (10)	0.0087 (10)	0.0028 (9)
C16	0.0331 (13)	0.0194 (10)	0.0308 (13)	0.0044 (9)	0.0181 (10)	0.0044 (9)
C17	0.0236 (11)	0.0172 (10)	0.0316 (13)	0.0004 (8)	0.0113 (9)	−0.0014 (9)
C18	0.0185 (10)	0.0152 (9)	0.0227 (11)	0.0021 (8)	0.0092 (8)	−0.0027 (8)
C19	0.0148 (10)	0.0212 (10)	0.0202 (11)	−0.0021 (8)	0.0073 (8)	−0.0031 (8)
C20	0.0216 (11)	0.0228 (11)	0.0307 (13)	−0.0033 (9)	0.0086 (9)	−0.0087 (9)
C21	0.0229 (11)	0.0378 (13)	0.0276 (13)	−0.0032 (10)	0.0015 (9)	−0.0167 (10)
C22	0.0231 (12)	0.0412 (13)	0.0218 (12)	0.0024 (10)	−0.0015 (9)	−0.0063 (10)
C23	0.0209 (11)	0.0257 (11)	0.0241 (12)	0.0025 (9)	0.0019 (9)	−0.0006 (9)
C24	0.0138 (10)	0.0202 (10)	0.0196 (11)	−0.0008 (8)	0.0038 (8)	−0.0046 (8)
C25	0.0234 (11)	0.0275 (11)	0.0188 (11)	0.0003 (9)	0.0023 (9)	−0.0057 (9)
C26	0.0213 (11)	0.0268 (10)	0.0159 (11)	−0.0005 (9)	0.0029 (8)	−0.0013 (8)
O5	0.0236 (8)	0.0247 (8)	0.0291 (9)	−0.0022 (6)	0.0034 (7)	−0.0016 (6)
C27	0.0162 (10)	0.0234 (10)	0.0268 (12)	0.0065 (9)	0.0039 (9)	−0.0008 (9)
C28	0.0210 (11)	0.0245 (11)	0.0319 (13)	0.0012 (9)	0.0069 (9)	−0.0014 (9)
C29	0.0257 (12)	0.0403 (13)	0.0255 (13)	−0.0010 (10)	0.0017 (10)	0.0027 (10)

### Geometric parameters (Å, º)

**Table:**

P1—N3	1.5675 (17)	C10—H10	0.9500
P1—N1	1.5837 (17)	C11—C12	1.382 (3)
P1—O1	1.5965 (14)	C11—H11	0.9500
P1—O2	1.6016 (14)	C13—C14	1.385 (3)
P2—N2	1.5665 (17)	C13—C18	1.388 (3)
P2—N1	1.5890 (17)	C14—C15	1.388 (3)
P2—O4	1.5940 (14)	C14—H14	0.9500
P2—O3	1.6041 (14)	C15—C16	1.387 (3)
P3—N2	1.6024 (17)	C15—H15	0.9500
P3—N3	1.6171 (17)	C16—C17	1.390 (3)
P3—N4	1.6471 (17)	C16—H16	0.9500
P3—N5	1.6514 (18)	C17—C18	1.402 (3)
O1—C1	1.407 (2)	C17—H17	0.9500
O2—C12	1.410 (2)	C18—C19	1.488 (3)
O3—C13	1.407 (2)	C19—C24	1.396 (3)
O4—C24	1.400 (2)	C19—C20	1.401 (3)
N4—C25	1.461 (3)	C20—C21	1.388 (3)
N4—H4N	0.82 (3)	C20—H20	0.9500
N5—C26	1.473 (3)	C21—C22	1.388 (4)
N5—H5N	0.83 (3)	C21—H21	0.9500
C1—C2	1.384 (3)	C22—C23	1.388 (3)
C1—C6	1.398 (3)	C22—H22	0.9500
C2—C3	1.386 (3)	C23—C24	1.381 (3)
C2—H2	0.9500	C23—H23	0.9500
C3—C4	1.389 (3)	C25—C26	1.526 (3)
C3—H3	0.9500	C25—H25A	0.9900
C4—C5	1.390 (3)	C25—H25B	0.9900
C4—H4	0.9500	C26—H26A	0.9900
C5—C6	1.402 (3)	C26—H26B	0.9900
C5—H5	0.9500	O5—C27	1.220 (3)
C6—C7	1.484 (3)	C27—C29	1.496 (3)
C7—C12	1.389 (3)	C27—C28	1.498 (3)
C7—C8	1.406 (3)	C28—H28A	0.9800
C8—C9	1.381 (3)	C28—H28B	0.9800
C8—H8	0.9500	C28—H28C	0.9800
C9—C10	1.388 (3)	C29—H29A	0.9800
C9—H9	0.9500	C29—H29B	0.9800
C10—C11	1.386 (3)	C29—H29C	0.9800
			
N3—P1—N1	119.36 (9)	C11—C12—O2	118.47 (18)
N3—P1—O1	104.64 (8)	C7—C12—O2	118.17 (17)
N1—P1—O1	111.37 (8)	C14—C13—C18	123.09 (19)
N3—P1—O2	113.28 (8)	C14—C13—O3	118.37 (18)
N1—P1—O2	105.03 (8)	C18—C13—O3	118.46 (18)
O1—P1—O2	101.90 (7)	C13—C14—C15	118.6 (2)
N2—P2—N1	119.30 (9)	C13—C14—H14	120.7
N2—P2—O4	105.18 (8)	C15—C14—H14	120.7
N1—P2—O4	110.65 (8)	C16—C15—C14	120.2 (2)
N2—P2—O3	113.07 (9)	C16—C15—H15	119.9
N1—P2—O3	105.59 (8)	C14—C15—H15	119.9
O4—P2—O3	101.71 (7)	C15—C16—C17	120.2 (2)
N2—P3—N3	112.12 (9)	C15—C16—H16	119.9
N2—P3—N4	112.47 (9)	C17—C16—H16	119.9
N3—P3—N4	113.39 (9)	C16—C17—C18	120.9 (2)
N2—P3—N5	113.64 (9)	C16—C17—H17	119.6
N3—P3—N5	109.59 (9)	C18—C17—H17	119.6
N4—P3—N5	94.47 (9)	C13—C18—C17	117.1 (2)
C1—O1—P1	123.88 (13)	C13—C18—C19	121.14 (18)
C12—O2—P1	116.68 (12)	C17—C18—C19	121.76 (19)
C13—O3—P2	116.39 (12)	C24—C19—C20	116.8 (2)
C24—O4—P2	124.52 (12)	C24—C19—C18	122.21 (18)
P1—N1—P2	117.77 (11)	C20—C19—C18	120.98 (19)
P2—N2—P3	123.95 (11)	C21—C20—C19	121.5 (2)
P1—N3—P3	123.96 (11)	C21—C20—H20	119.3
C25—N4—P3	110.45 (14)	C19—C20—H20	119.3
C25—N4—H4N	117.0 (17)	C22—C21—C20	119.9 (2)
P3—N4—H4N	117.8 (18)	C22—C21—H21	120.0
C26—N5—P3	111.99 (14)	C20—C21—H21	120.0
C26—N5—H5N	118.6 (17)	C21—C22—C23	119.9 (2)
P3—N5—H5N	115.9 (17)	C21—C22—H22	120.1
C2—C1—C6	122.12 (19)	C23—C22—H22	120.1
C2—C1—O1	117.93 (18)	C24—C23—C22	119.3 (2)
C6—C1—O1	119.65 (18)	C24—C23—H23	120.3
C1—C2—C3	119.36 (19)	C22—C23—H23	120.3
C1—C2—H2	120.3	C23—C24—C19	122.56 (19)
C3—C2—H2	120.3	C23—C24—O4	116.64 (18)
C2—C3—C4	120.1 (2)	C19—C24—O4	120.46 (18)
C2—C3—H3	120.0	N4—C25—C26	103.74 (17)
C4—C3—H3	120.0	N4—C25—H25A	111.0
C3—C4—C5	120.1 (2)	C26—C25—H25A	111.0
C3—C4—H4	120.0	N4—C25—H25B	111.0
C5—C4—H4	120.0	C26—C25—H25B	111.0
C4—C5—C6	120.97 (19)	H25A—C25—H25B	109.0
C4—C5—H5	119.5	N5—C26—C25	104.18 (17)
C6—C5—H5	119.5	N5—C26—H26A	110.9
C1—C6—C5	117.38 (19)	C25—C26—H26A	110.9
C1—C6—C7	121.47 (18)	N5—C26—H26B	110.9
C5—C6—C7	121.13 (18)	C25—C26—H26B	110.9
C12—C7—C8	116.76 (18)	H26A—C26—H26B	108.9
C12—C7—C6	121.56 (18)	O5—C27—C29	120.8 (2)
C8—C7—C6	121.68 (18)	O5—C27—C28	122.0 (2)
C9—C8—C7	120.7 (2)	C29—C27—C28	117.21 (19)
C9—C8—H8	119.6	C27—C28—H28A	109.5
C7—C8—H8	119.6	C27—C28—H28B	109.5
C8—C9—C10	120.73 (19)	H28A—C28—H28B	109.5
C8—C9—H9	119.6	C27—C28—H28C	109.5
C10—C9—H9	119.6	H28A—C28—H28C	109.5
C11—C10—C9	119.85 (19)	H28B—C28—H28C	109.5
C11—C10—H10	120.1	C27—C29—H29A	109.5
C9—C10—H10	120.1	C27—C29—H29B	109.5
C12—C11—C10	118.6 (2)	H29A—C29—H29B	109.5
C12—C11—H11	120.7	C27—C29—H29C	109.5
C10—C11—H11	120.7	H29A—C29—H29C	109.5
C11—C12—C7	123.33 (18)	H29B—C29—H29C	109.5
			
N3—P1—O1—C1	−151.76 (15)	C5—C6—C7—C12	137.4 (2)
N1—P1—O1—C1	77.97 (16)	C1—C6—C7—C8	136.4 (2)
O2—P1—O1—C1	−33.56 (16)	C5—C6—C7—C8	−41.9 (3)
N3—P1—O2—C12	54.75 (16)	C12—C7—C8—C9	0.0 (3)
N1—P1—O2—C12	−173.32 (14)	C6—C7—C8—C9	179.33 (19)
O1—P1—O2—C12	−57.08 (15)	C7—C8—C9—C10	0.2 (3)
N2—P2—O3—C13	52.84 (16)	C8—C9—C10—C11	−0.4 (3)
N1—P2—O3—C13	−175.00 (13)	C9—C10—C11—C12	0.5 (3)
O4—P2—O3—C13	−59.42 (15)	C10—C11—C12—C7	−0.3 (3)
N2—P2—O4—C24	−148.34 (15)	C10—C11—C12—O2	−178.18 (18)
N1—P2—O4—C24	81.56 (17)	C8—C7—C12—C11	0.1 (3)
O3—P2—O4—C24	−30.24 (17)	C6—C7—C12—C11	−179.23 (19)
N3—P1—N1—P2	−1.48 (16)	C8—C7—C12—O2	177.90 (17)
O1—P1—N1—P2	120.63 (11)	C6—C7—C12—O2	−1.4 (3)
O2—P1—N1—P2	−129.84 (11)	P1—O2—C12—C11	−103.17 (18)
N2—P2—N1—P1	16.39 (16)	P1—O2—C12—C7	78.9 (2)
O4—P2—N1—P1	138.55 (10)	P2—O3—C13—C14	−103.07 (18)
O3—P2—N1—P1	−112.16 (11)	P2—O3—C13—C18	80.20 (19)
N1—P2—N2—P3	−17.32 (17)	C18—C13—C14—C15	−0.4 (3)
O4—P2—N2—P3	−142.15 (12)	O3—C13—C14—C15	−176.97 (17)
O3—P2—N2—P3	107.71 (12)	C13—C14—C15—C16	0.8 (3)
N3—P3—N2—P2	2.48 (16)	C14—C15—C16—C17	−0.6 (3)
N4—P3—N2—P2	131.67 (12)	C15—C16—C17—C18	−0.1 (3)
N5—P3—N2—P2	−122.48 (13)	C14—C13—C18—C17	−0.3 (3)
N1—P1—N3—P3	−14.21 (17)	O3—C13—C18—C17	176.31 (17)
O1—P1—N3—P3	−139.60 (11)	C14—C13—C18—C19	−179.62 (18)
O2—P1—N3—P3	110.26 (12)	O3—C13—C18—C19	−3.0 (3)
N2—P3—N3—P1	13.44 (16)	C16—C17—C18—C13	0.5 (3)
N4—P3—N3—P1	−115.26 (13)	C16—C17—C18—C19	179.83 (19)
N5—P3—N3—P1	140.61 (12)	C13—C18—C19—C24	−41.5 (3)
N2—P3—N4—C25	138.76 (14)	C17—C18—C19—C24	139.2 (2)
N3—P3—N4—C25	−92.72 (16)	C13—C18—C19—C20	139.6 (2)
N5—P3—N4—C25	20.88 (16)	C17—C18—C19—C20	−39.7 (3)
N2—P3—N5—C26	−112.97 (15)	C24—C19—C20—C21	−0.2 (3)
N3—P3—N5—C26	120.73 (15)	C18—C19—C20—C21	178.7 (2)
N4—P3—N5—C26	3.95 (16)	C19—C20—C21—C22	0.8 (3)
P1—O1—C1—C2	−117.03 (18)	C20—C21—C22—C23	−0.7 (4)
P1—O1—C1—C6	69.1 (2)	C21—C22—C23—C24	−0.1 (3)
C6—C1—C2—C3	0.1 (3)	C22—C23—C24—C19	0.7 (3)
O1—C1—C2—C3	−173.67 (18)	C22—C23—C24—O4	−172.71 (19)
C1—C2—C3—C4	0.2 (3)	C20—C19—C24—C23	−0.5 (3)
C2—C3—C4—C5	−0.7 (3)	C18—C19—C24—C23	−179.51 (19)
C3—C4—C5—C6	1.0 (3)	C20—C19—C24—O4	172.62 (18)
C2—C1—C6—C5	0.2 (3)	C18—C19—C24—O4	−6.3 (3)
O1—C1—C6—C5	173.83 (17)	P2—O4—C24—C23	−118.86 (18)
C2—C1—C6—C7	−178.11 (19)	P2—O4—C24—C19	67.6 (2)
O1—C1—C6—C7	−4.5 (3)	P3—N4—C25—C26	−37.8 (2)
C4—C5—C6—C1	−0.7 (3)	P3—N5—C26—C25	−25.7 (2)
C4—C5—C6—C7	177.58 (19)	N4—C25—C26—N5	38.3 (2)
C1—C6—C7—C12	−44.4 (3)		

### Hydrogen-bond geometry (Å, º)

**Table:**

*D*—H···*A*	*D*—H	H···*A*	*D*···*A*	*D*—H···*A*
N4—H4*N*···O5	0.82 (3)	2.22 (3)	2.991 (2)	158 (2)
N5—H5*N*···N3^i^	0.83 (3)	2.53 (3)	3.285 (2)	151 (2)

Symmetry code: (i) −*x*+2, −*y*+1, −*z*+1.
